# Probabilistic Risk Analysis to Assess Dietary Exposure to Aluminum in the Taiwanese Population

**DOI:** 10.3390/ijerph18031099

**Published:** 2021-01-26

**Authors:** Shu-Han You, Szu-Chieh Chen, Chin-Hsin Lin, Yen-Chu Chen

**Affiliations:** 1 Institute of Food Safety and Risk Management, National Taiwan Ocean University, Keelung City 20224, Taiwan; shyou@mail.ntou.edu.tw; 2 Department of Public Health, Chung Shan Medical University, Taichung 40201, Taiwan; fysh011154@gmail.com (C.-H.L.); qrevalf64@gmail.com (Y.-C.C.); 3 Department of Family and Community Medicine, Chung Shan Medical University Hospital, Taichung 40201, Taiwan

**Keywords:** estimated weekly intake, risk assessment, consumption, Taiwan, aluminum

## Abstract

Aluminum (Al) exposure at human dietary levels raises health concerns, yet little is known about the Al exposure from the Taiwanese diet. The amount of aluminum (Al)-containing food consumption in the Taiwanese total diet is increasing, which contributes to the total diet consumption., which raises the health concerns. In this study, we aim to assess estimated weekly intake (EWI) and the percentage of provisional tolerable weekly intake (%PTWI) of the dietary exposure to Al in different age-sex groups. We also applied probabilistic risk analyses to quantify the parameters’ uncertainty by focusing on the distribution function for the Al concentration in food, consumption rate, and body weight in specific age groups. Results indicated that the EWIs declined with increasing age after 6-years old (7–12 > 13–15 > 16–18 > 19–64 > 65+). Results indicated that the EWIs gradually declined after 6-year of age. The EWIs of Al-rich food in cake + waffle, kelp, snacks, and bread contributed 20%, 17%, 17%, and 11%, respectively, to the total EWIs, corresponding with the much higher consumption rates for these four foods. The 75th percentile of EWIs for the children aged 34–6 years had a %PTWI valued at over 100%, indicating a potential risk of Al intake via dietary exposure. Our findings show that there is a concern about the consumption of Al-rich foods for children in Taiwan.

## 1. Introduction

Aluminum (Al) is the third most abundant element on Earth and is widely used in human daily life. Routine ingestion of Al compounds can lead to significant accumulations in the organs and aging-tissues in normal people and in rat [1,2]. Moreover, research has shown that Al exposure at human dietary levels promotes vascular dysfunction and increases blood pressure in rats [3]. The accumulation of Al compounds in the human body has been associated with neurological disorders [4,5,6], skeletal system, hematopoietic system, and osteomalacia [7], and there is also an ongoing debate about the role of Al in Alzheimer’s disease and dialysis encephalopathy [8,9,10,11].

In 1989, the Joint FAO/WHO Expert Committee on Food Additives (JECFA) established the provisional tolerable weekly intake (PTWI) for Al of 7.0 mg per kg body weight (mg/kg-bw) [12]. JECFA re-evaluated the safety of Al and lowered the PTWI to 1.0 mg/kg-bw in 2007 because of the observed effects on the reproductive system and developing nervous system in experimental animals [13]. However, the JECFA later revised the PTWI to 2.0 mg/kg-bw in 2011 [14] as a result of new bioavailability and toxicology data in the rat [15]. This PTWI is applied to Al compounds in food, including Al-containing food additives [14].

For the past years, researchers have focused on measuring and assessing the dietary exposure to Al from processed foods/raw materials/food additives [16], wheat flour/puffed products [17], food and kitchenware [18], and high Al-containing food additives [19]. National-specific studies combined with food frequency questionnaires (FFQ), large sample sizes, and multiple age groups for food have also been mentioned in countries [20,21,22,23,24]. In Japan, the average weekly dietary Al exposure from processed food for young children, children, youths, and adults is estimated to be 0.86, 0.45, 0.35, and 0.30 mg/kg-bw/wk, respectively. The %PTWI for all age groups is 43.1, 22.4, 17.6, and 15.1%, respectively. Only the highest consumer Al exposure value (>P95) of the young children group exceeded the PTWI [22]. In the Spain, González-Weller et al. reported intakes of 10.171 mg/day, slightly higher than the PTWI [20]. Furthermore, in China, Ma et al. reported an average intake of 1.795 mg/kg-bw/wk, not exceeding the PTWI. However, it was found that the dietary exposure to Al for 42.6% of children aged 4–6 years exceeded the PTWI [23]. Besides this, Filippini et al. indicated that the intake for the Italian population does not exceed the PTWI. A large-scale food consumption survey and multiple food measures from Al exposure could provide valuable information for whole human populations [24].

To our knowledge, only a few papers have been published regarding the investigation of Al concentration in food and the exposure assessment for human health in Taiwan. Yeh et al. performed Al exposure assessments on imported snacks and candies in two age groups: 4–6 and 19–64 years of age, however, other known Al-rich food items were not considered in the dietary intake assessment [25].

Thus, the aims of this study were: (1) to assess estimated weekly intake (EWI) and %PTWI of dietary exposure to Al in different age-sex groups in Taiwan; (2) to consider the uncertainty of parameters by focusing on the distribution function for the Al concentration in food, consumption rate, and body weight in specific age groups. A Monte Carlo (MC) simulation is used for probabilistic risk analyses, and (3) to consider the Al-rich food and high consumption food items both that could fully consider the total diet exposure for the Taiwanese population.

## 2. Material and Methods

### 2.1. Aluminum Concentration in Food

For comprehensively estimating aluminum (Al) concentration in food for all populations, the Al-rich food and high consumption food items were considered in this study. We adopted the assessment method for Al concentration in food from Zhou in order to assess the risk of dietary intake of Al-rich foods in Taiwan [26]. We also adopted the high consumption food items containing Al from Yeh et al. [25], Jiang et al. [27], and Yang et al. [21].

A total of 150 food samples were analyzed by Zhou. Twelve categories were adopted: steamed pastries (N = 17), steamed sponge cakes (N = 15), breads (N = 13), cakes (N = 12), waffles (N = 25), biscuits (N = 14), fried breadsticks (N = 14), snacks (N = 13), salted jellyfish body (N = 10), kelps (N = 10), sugar-coated products (N = 2), and green bean noodles (N = 5). The 12 categories were mainly grouped by main ingredient, processing method, or nutritional composition, and are regularly consumed by Taiwanese people [26].

In Yeh et al.’s study [25] that examined the Al contents of imported candies and snack foods in Taiwan a high daily intake of chocolates and candies among children (3–6 years of age) was observed. They analyzed a total of 67 samples, including five chewing gums, seven dried fruits, 13 chocolates, two jellies, two dried squid pieces, and 38 candies. Thus work also provided detailed information about the methodology, including the equipment used, sample collection methods, pretreatment and method validation, precision, accuracy, ND and detection limit of quantification (LOQ = 1.53 μg/g).

However, after reviewing the published data and 17 food groups in Taiwan National Food Consumption Database (TNFCD) [28], we found that data for Al concentrations associated with the major food groups consumed in the Taiwanese region still lacked part of the groups such as rice, milk, eggs, vegetables, drinking water, etc. Complementary to Al dietary exposure assessment in Taiwan, the Al content for an additional eight food consumption items were adopted from Jiang et al. [27], and the Al in the drinking water item was adopted 0.052 ppm from Yang et al.’s [21] study on South China to gather a range of foods representative of the diet and as close as possible to the consumers’ dietary habits to accurately reflect dietary exposure to Al in Taiwan.

### 2.2. Food Consumption Data

The Taiwan National Food Consumption Database (TNFCD) was established by the Taiwan government in 2012 and was based on the previous Nutrition and Health Survey in Taiwan (NAHSIT) 2005–2012 and NAHSIT 2013–2016. The NAHSIT 2005–2012 utilized a 24-h dietary recall, and the target populations were preschoolers (2005–2008), elementary school students (2005–2008), and junior and senior-high-school students (2010–2011). In the NAHSIT 2013–2016, sampling was extended from the previous sampling design of the NAHSIT 2005–2012. The website of TNFCD is convenient for adopting food consumption data in different age-sex groups [29]. Moreover, the database is the significant information source for risk assessment of food contaminants.

The food consumption information utilized in this study were obtained from the 2017 TNFCD (http://tnfcds.cmu.edu.tw/index.php?action=index). The selected intake data have to be combined with the estimated Al concentration in 21 food items. The sample size of this study was 7204 (3555 males and 3694 females), and the eight age groups were 0–3, 4–6, 7–12, 13–15, 16–18, 19–64, 65+ years of age, and childbearing age (19–45 years for women only). A statistical analysis of the consumption rate (g/day), stratified by sex, including minimum, maximum, mean, standard error (SE), and standard deviation (SD) was performed for the 21 food items. Consumption rate data were available for the general population and for consumers only (N = 2951 with 1266 males and 1685 females). However, parts of the food consumption data were estimated from small sample sizes (N < 5). Therefore, in order to avoid large deviations in the estimation values, only the mean consumption rate with SD for the general population exposure to the food items was used.

### 2.3. Dietary Exposure Assessment (EWI and %PTWI)

The estimated weekly intake (EWI) is defined as the weekly average exposure dose (mg/kg-bw/wk) and was stratified according to age-sex groups. The EWI of Al for each group was calculated by multiplying the fitted distribution of Al concentration by the fitted distribution of daily consumption rates of the corresponding food item within each age-sex group, and then each mean estimate was multiplied by 7 (days/week). A percentage of the EWI to the provisional tolerable weekly intake (PTWI) (2 mg/kg-bw/wk) was used to assess dietary exposure according to the Joint FAO/WHO Expert Committee on Food Additives (JECFA), it was then expressed as %PTWI, which is the tentative amount of Al that can be tolerated weekly and be calculated at an individual level in this study. A value less than 100% indicates that there may not be a concern for potential human health effects associated with Al exposure.

Hence, EWI and %PTWI can be expressed as:

(1) $$EWI_{ijk}=\frac{C_{i}\;\times \;IR_{ijk}\;}{BW_{jk}}\;\times 0.001\times 7$$

(2) $$\%PTWI_{ijk}=\frac{EWI_{ijk}}{PTWI}\;\times 100\%$$

where *C_i_* (mg/kg) refers to the concentration of Al in the 21 food items, and *i* = 1–21 refers to the Al concentrations in steamed pastry, steamed sponge cake, waffle, fried breadstick, salted jellyfish body, kelp, bread cake, biscuits, snacks, sugar-coated products, green bean noodle, rice, milk, eggs, meat, vegetables, fish and seafood, beans, fruit, and drinking water, respectively; *IR_ijk_* (g/day) refers to the amount of each food item consumed according to sex (j = 1 (male), 2 (female)) and age group (*k* = 1–8), where *k* refers to the age groups classes of 0–3, 4–6, 7–12, 13–15, 16–18, 19–64, 65+ years of age and childbearing age (19–45 years for women only), respectively, whilst *BW_jk_* (kg) refers to the bodyweight of the specific age-sex groups. In Equation (1), the coefficients of 0.001 and 7 are the unit conversion values that can be used for converting one day to a week, respectively.

### 2.4. Monte Carlo Simulation

A Monte Carlo (MC) simulation was used to characterize the uncertainties of parameters and exposure scenarios for the EWI estimation among age-sex groups. All input parameters were the Al concentration in food (*C*), and the consumption rate (*IR*), and body weight (*BW*). The uncertainty of exposure scenario was eight age groups classes of 0–3, 4–6, 7–12, 13–15, 16–18, 19–64, 65+ years of age and childbearing age (19–45 years for women only). These data were fitted into the most appropriate or generated pre-defined probability distributions based on past experience or historical researches by using the Crystal Ball software (Version 2000.2, Decisioneering Inc., Denver, CO, USA). A log-normal distribution model was assumed to describe the input parameters of *C* and *IR*, whereas a normal distribution was used for *BW*. The *BW* for all age-sex groups is listed in Table S1. The two reasons for the assumed distribution model are that: (i) raw data fit the log-normal distribution according to the Kolmogorov-Smirnov test; and (ii) The previous studies [25,30] have mentioned the concentration in food available with which to define a log-normal distribution.

Moreover, to explicitly quantify the uncertainty and variability of the data, a MC simulation was performed with 100,000 iterations via the simple random sampling method (stability condition) to obtain the result of robust uncertainty analysis. The sampling data was used to construct its corresponding 95% confidence interval as the uncertainty range. The process of repeatedly sampling from probability distributions derived the distribution of outcomes. The MC simulation was implemented using Crystal Ball software.

Sensitivity analysis was used to know the magnitude of the variance or uncertainty of the assumption parameter and the direction of the correlation of the assumption parameter with the EWI. Sensitivity analysis was performed with Crystal Ball by computing Spearman rank correlation coefficients between the assumption parameter and the EWI. Rank correlation of each assumption parameter shows sensitivities as rank correlation coefficients that range from −1 to +1. The assumption parameter with a high correlation coefficient represents a more significant impact on the EWI. Positive coefficients mean that an increase in the parameter is associated with an increase in the EWI. Negative coefficients mean that the parameters are inversely related.

## 3. Results

### 3.1. Aluminum Concentration in Different Food Items

**Figure 1:**
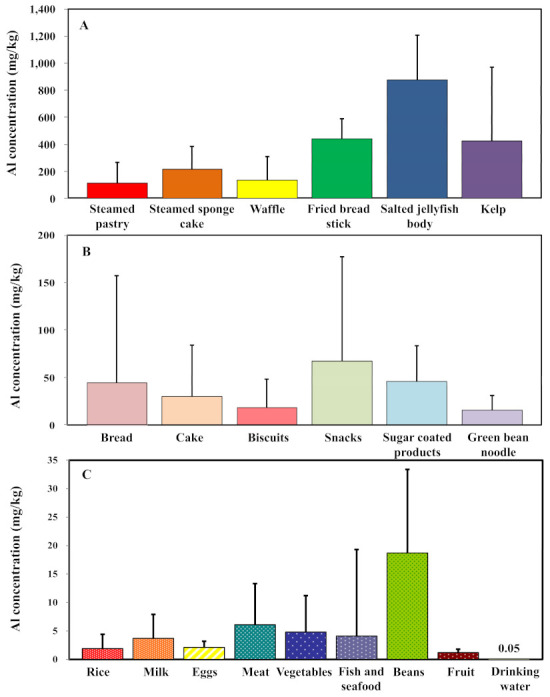
(**A**,**B**) Mean aluminum concentrations for 12 aluminum-rich food items (mg/kg) in Taiwanese regions (adopted from Zhou [25] and Yeh et al. [26]. The bars indicate the standard deviation (SD) for each food category. (**C**) Mean aluminum concentration of nine additional food consumption items (mg/kg) (adopted from Jiang et al. [21] and Yang et al. [27].

The aluminum (Al) concentrations found in 21 food items are shown in Figure 1. All concentrations were adopted from Zhou [26], Yeh et al. [25], Jiang et al. [27], and Yang et al. [21]. The original statistical descriptions are shown in Table 1 and Table 2. The results indicate that the salted jellyfish body contained the highest amount of Al, with a mean concentration and standard deviation (SD) of 876.6 ± 328.2 mg/kg. The Al concentrations (mean ± SD) found in fried breadsticks, kelp and steamed sponge cakes were 440.9 ± 150.1, 424.5 ± 547.1, and 217.3 ± 169 mg/kg, respectively (Figure 1A, Table 1). Al was also detected in rice, milk, and eggs, but the levels were relatively low at 1.9 ± 2.5, 3.7 ± 4.2, and 2.1 ± 1.1 mg/kg, respectively (Figure 1C, Table 1). The median concentration of Al in drinking water was 0.052 mg/kg (Figure 1C, Table 1).

### 3.2. Food Consumption Data across Age-Sex Subgroups

The consumption rate (g/day) of 12 Al-rich food items for the different age-sex groups sourced from the 2017 TNFCD is illustrated in Figure S1. Each column expresses the total consumption rate according to specific age-sex groups. Consumption rates were higher for 4–18-year-olds (range from 55–65 g/day) compared to the other groups’ intake. In contrast, the 65+ (F) age group had the lowest intake of all the 12 Al-rich foods (about 25 g/day), and that for childbearing women is 48 g/day.

**Figure 2:**
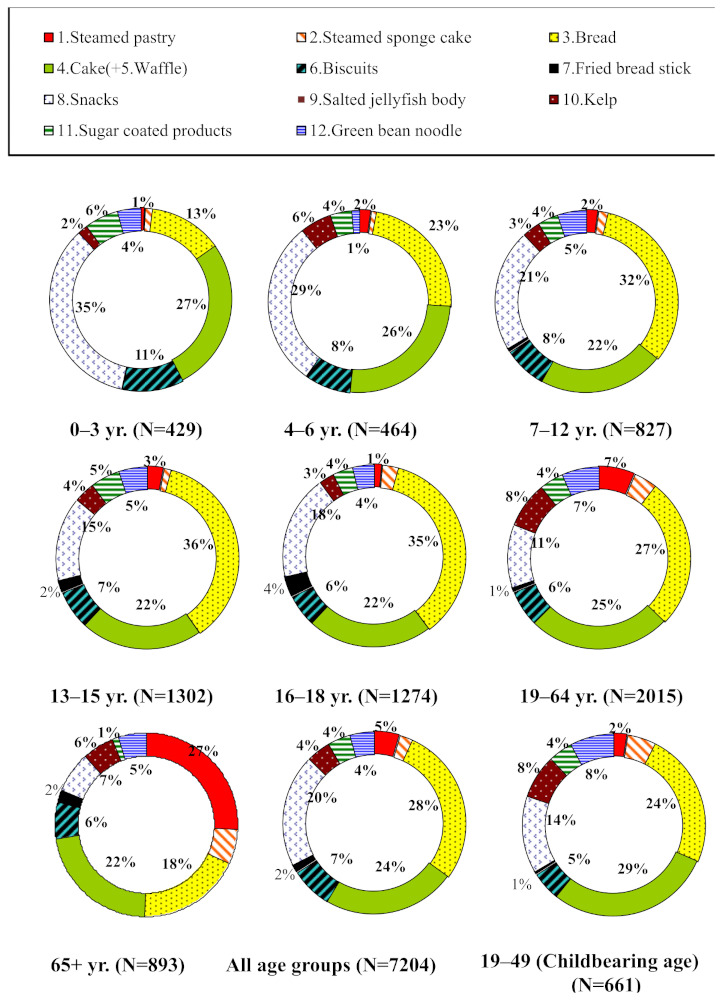
The contribution percentage (%) of the mean consumption rate for 12 aluminum-rich food items in specific age groups, the whole population, and women of childbearing age, respectively.

The consumption of bread, cake + waffle, and snacks across all the age-sex groups contributed the highest percentage of Al to dietary intake and ranged from 13–36%, 22–27%, and 7–35% across the different age groups, respectively, as shown in Figure 2. On average, for all age groups, the top three food items with the highest Al concentration of all the 12 Al-rich food items were bread, cake + waffle, and snacks, which contributed 28%, 24%, and 20%, respectively (Figure 2). A detailed dataset of dietary intake is given in Table S2. Because the highest Al concentration was contained in salted jellyfish body, fried breadstick, and kelp, Table S2 gives the consumption rates (g/day) for these three food items in the different age-sex groups. On average, the highest intakes of these three food items were estimated to be 0.03 g/day for 6–65+ years, 2.20 (F)/2.50 (M) g/day for 16–18 years, 3.99 g/day (M, 4–6 years), and 3.61 g/day (F, 19–64 years).

**Figure 3:**
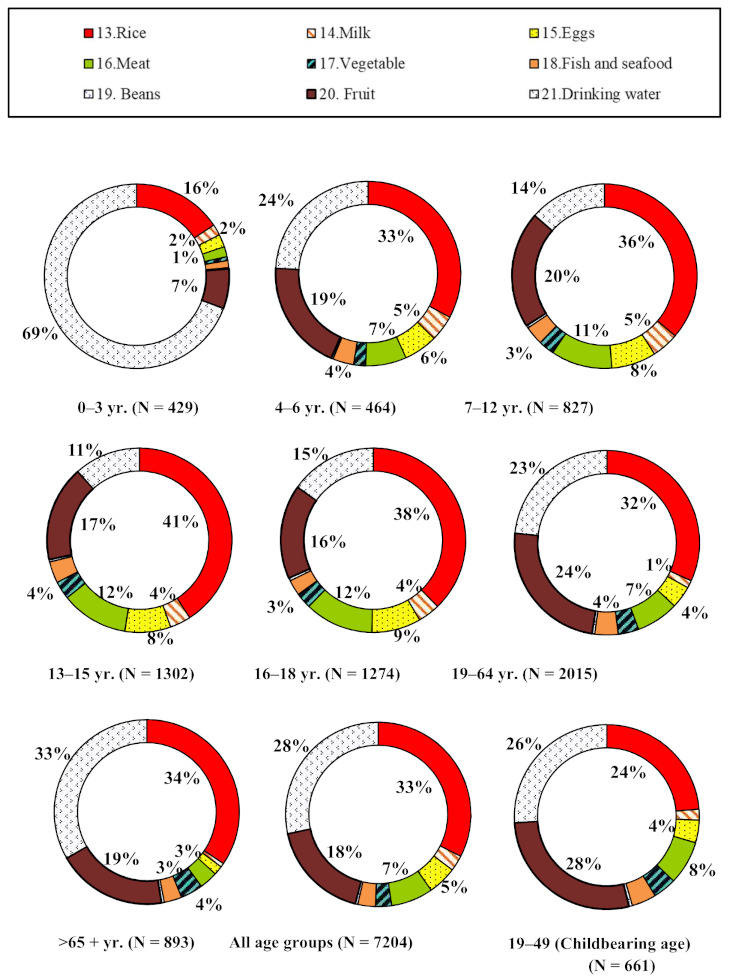
The contribution percentage (%) of the mean consumption rate for 9 additional food consumption items in specific age groups, the whole population, and women of childbearing age, respectively.

On average, for all age groups, the top four highest food items consumed by Taiwanese people were rice, drinking water, fruit, and meat, which contributed 33%, 28%, 18%, and 7%, respectively, for the remaining nine food items (Figure 3). Because the highest Al concentration was contained in beans and meat (Figure 1C), the highest intakes of these two food items were estimated to be 3.81 g/day (F, 19–64 years), 5.26 g/day (M, 65+ years), 85.50 g/day (M, 16–18 years) and 57.78 g/day (F, 13–15 years).

**Figure 4:**
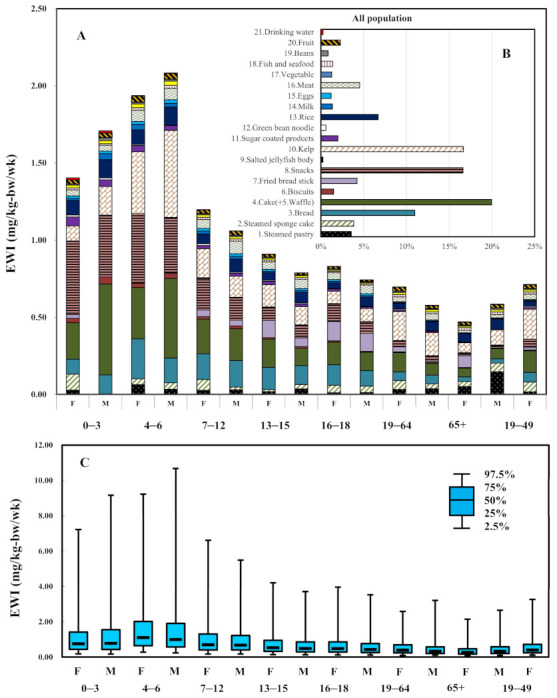
(**A**) Estimated weekly intake (EWI, mg/kg-bw/wk) of 21 food items for all age-sex groups. (**B**) Contribution percentage (%) for the whole population, excluding the age groups from 19–45 years (children bearing age), among 21 food items (**C**) Box-whisker plot with 95% confidence interval of EWI estimations.

**Figure 5:**
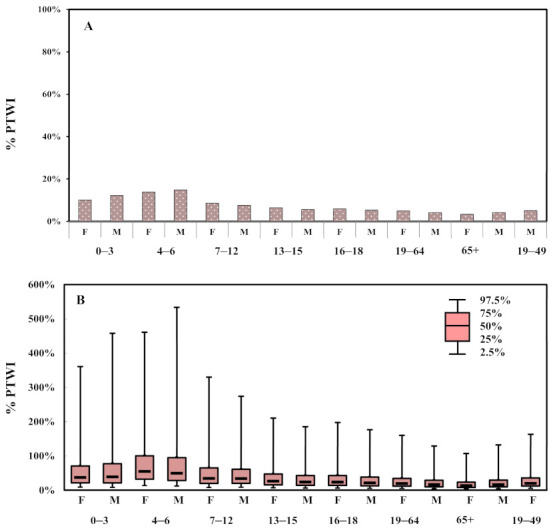
(**A**) Estimated % PTWI for all age-sex groups. (**B**) Box-whisker plot shows the 95% confidence interval for % PTWI estimations.

The intake of the additional nine food consumption items is also presented in Figure 4 and Figure 5, and Table S3. The results show that consumption rates of Al in the nine food items were higher for males than females, especially in the 13–15, 16–18, 19–64, and 65+ years age groups. Of the nine food items the three with high consumption rates were rice, meat, and drinking water (Figure S2).

### 3.3. Risk Assessment of Dietary Exposure to Aluminum

The estimated weekly intake (EWI) of Al exposure across all the age-sex groups is shown in Figure 4. Across all age groups, the EWI was 1.071 mg/kg-bw/wk. The EWIs were higher for the 0–3, and 4–6 years age groups compared with the other groups and ranged from 1.404–2.083 mg/kg-bw/wk. Whilst, the EWIs declined with increasing age (7–12 > 13–15 > 16–18 > 19–64 > 65+) (Figure 4A). The contributions of each Al-rich food item to the EWI were 20% for cake + waffle, 17% for kelp, 17% for snacks, and 11% for bread, respectively (Figure 4B), which corresponds with the much higher consumption rates for these four foods. Detailed EWIs for the age-sex groups are listed in Table 2. Based on the sensitivity analysis, the results indicated that the intake rates of cake and waffle, kelp, and snacks showed a positive correlation coefficient and reflected a direct relationship between Al levels and the EWIs. Otherwise, the parameter of *BW* played a negative correlation coefficient and reflected an inverse relationship between *BW* and the EWIs (Figures S3 and S4). For dietary exposure to Al in the Taiwanese population, the results of sensitivity analysis of *BW* show that the correlation coefficients of assumption parameters with EWIs for the age groups were ranged from −0.380 to −0.212 and −0.373 to −0.144 for females and males, respectively. The body weight factor may have an effect to prevent dietary exposure to Al.

The %PTWI estimations for each age-sex group are illustrated in Figure 5. The EWIs for the 0–3, and 4–6 year age groups were estimated to be 1.404 (F), 1.708 (M), 1.937 (F), 2.083 (M) mg/kg-bw/wk, respectively, which accounted for 10%, 12%, 14% and 15% of the PTWI, respectively (Figure 5A). The 75th percentile of EWIs for the aged 4–6 years had a %PTWI valued at over 100%, indicating a potential risk of Al intake via dietary exposure for this group (Figure 5B). Figure 4C and Figure 5B illustrate the 95% confidence interval for the EWI and %PTWI estimations. A box-and-whisker plot represents the selected percentiles of the EWIs of Al intake according to age and gender.

## 4. Discussion

This study conducts the risk assessment for human exposure to 12 Aluminum (Al) -rich food items and nine additional food consumption items for the Taiwanese population. We used the consumption rate from the latest dietary survey in a relatively large study population size from all age groups in Taiwan (N = 2951 with 1266 males and 1685 females). The average dietary Al exposure level in the Taiwan population is 1.071 mg/kg-bw/wk, which is lower than the provisional tolerable weekly intake (PTWI). Age-sex analyses suggested that the children (0–3 and 4–6 years) intake high-level Al and ranged from 1.404–2.083 mg/kg-bw/wk. The risk decreased with aging. In addition, the cake + waffle, kelp, snacks, and bread items were major contributors to Al exposure in the population.

These findings are similar to the results from a study in China [17]. Guo at al. [17] conducted a study on Al exposure in food by obtaining 400 food samples from Shanghai, China and measuring the Al concentration in each sample between 2011 and 2013. The highest levels of Al were observed in children aged 2–6 years (1.88 mg/kg-bw/wk). Yang et al. [21] collected 21,792 food samples from 853 subjects located in Shenzhen, China, to examine their exposure to Al-containing foods and asked the subjects to recall food substances ingested within the previous 24 h. The groups with the highest exposure to Al were children aged 0–2 and 3–13 years, with 3.356 mg/kg-bw/wk and 3.248 mg/kg-bw/wk, respectively.

However, Hayashi et al. collected 949 food samples for three consecutive days from 319 subjects in Japan during 2006–2010. The median 3-day average intake was calculated to be 0.2877 mg/kg-bw/wk [31]. Tietz et al. assessed the health risks resulting from total consumer exposure to Al. The mean dietary intake for the German adult aged 14–80 years of age range between 0.18 and 0.21 mg/kg-bw/wk [32]. On the other hand, available studies indicate that low dose of chronic exposure to Al compounds may have the potential to induce some behavioral changes, brain aging and neurodegeneration in experimental mice or rats [33,34]. These studies also suggested that further study will be needed to reevaluate the behavioral outcomes of oral intake of low dose of Al compounds.

The most common methods for dietary exposure to environmental and food contaminants are deterministic point estimates and probabilistic modeling [31]. Based on the Monte Carlo simulation, we set three parameters as probabilistic assumptions and to predict the uncertainty of the estimated weekly intake (EWI) and %PTWI by different age-sex groups. It indicates that most people exposure to Al levels lower than the PTWI, but the large variations of EWI and %PTWI were shown in Figure 4C and Figure 5B. The reason might cause by the standard deviation of Al concentration in food (Table 1 and Table 2) and the variations of consumption rate for the general population (Table S2). The uncertainties of input parameter for the EWI (Table S4). The variations of the residues of Al in food samples were likely due to a different amount of Al-containing food additives used by different manufactures. Thus, the high consumption rate for high Al-contaminant food still has to pay attention in further study (range from 75th percentile to 97.5th percentile of EWI and % PTWI).

The results of age-sex analyses suggest that the children (0–3 and 4–6 years) may have a high-level intake of Al. In the Taiwan National Food Consumption Database, among the consumption rates of the milk items consumed for the 0–3 years, the highest autism consumption rates were the infant formula (77.19 g/day), followed by wheat gluten (68.10) and whole milk (25.89), respectively. Although studies have documented Al content in infant formula could be an important source of exposure for the 0–3 years [32,33], in Taiwan, infant formula diet has not been recorded to cause any effects on health. On the other hand, Al-containing adjuvants for children in vaccines have been suspected of causing effects, which include neurotoxicity and the autism spectrum, however, findings of an evidence-based meta-analysis of case-controlled studies suggest that vaccinations are not associated with the development of autism [34]. Further studies could assess the potential health risks attributable to the Al concentration in infant formulas, especially in potentially vulnerable populations.

Some Chinese herbal medicines may be consumed for both medicinal pirposes and in traditional food dishes or products [35]. In Asia, herbal food product remedies are considered as traditional therapies. Moreover, some studies indicated that ginseng and dietary Chinese herbal products could have high Al concentrations [36,37,38]. Chen found that the Al concentration of *Angelica keiskei*, *Angelica sinensis* (Danggui in Mandarin), *Ligusticum chuanxiony* (chuanxiong in Mandarin), and *Panax ginseng* (ginseng in Mandarin) in Taiwan was 230–1170, 884, 956, and 196 ppm, respectively [36]. Based on the Taiwan National Food Consumption Database, for the age group of 19–64 years mean value of soup consumption containing Chinese herbal medicine is 3.21 g/day, and the maximum consumption is 548.97 g/day. If *Angelica sinensis* is consumed at 3.21 g/day in soup for 7 days and the Al concentration 884 mg/kg is adopted, the crude estimated weekly intake for 60 kg adult would be 0.33 mg/kg-bw/wk. Even in the low consumption rate, a relatively high concentration in dietary Chinese herbal products could make a significant contribution to Al exposure.

In this study, the value for Al in drinking water (0.052 ppm) was adopted from Yang et al. [24] for the exposure assessment. In a previous study, Chen and Liu found that the Al content for tap water was 1.241 ppm, which is higher than the mentioned value [39]. The adopted value might therefore not reflect the Al concentration in drinking water in Taiwan. The different water use behaviors were from tap water and drinking water in urban and rural cities. The water use behavior differences between urban and rural cities may affect exposure to Al in drinking water.

A previous study had been carried out to determine Al concentrations in young leaves from four major producing areas in Taiwan [40]. The Al concentrations in young leaves were between 570–1232 ppm. Chen and Liu examined the Al content in Oolong tea, which ranged from 1.46 and 1.10 ppm in the first and second infusion, respectively [39]. According to the Taiwan National Food Consumption Database, for the age group 19–65 years, the mean value for tea drink consumption is 376.11 g/day. Based on two studies [39,40], the crude estimated weekly intake for 60 kg adult would be 45.06 and 0.064 mg/kg-bw/wk for direct intake of young leaves and drinking Oolong tea, respectively. Moreover, the current studies of de Oliveira [41] and Tietz and Troisi [42] suggest that tea drinking could be a possible important source of Al exposure. Although drinking Al-contaminated tea is unlikely to be a significant safety concern by comparing the provisional tolerable weekly intake 2 mg/kg-bw/wk. From the conservative point of view, if people directly intake young leaves, they could have a potential health concerns.

The main dietary source was the use of Al-containing food additives [43,44]. At present, the use of Al-containing food additives in Taiwan follows the limits and specifications set by the Taiwan Food and Drug Administration (TFDA), which is the agency responsible for regulating expansion agents, quality improvers, colorants, thickeners and emulsifiers. The TFDA states that the use of Al-containing food additives should be based on need and restricts their use in fresh foods such as meat, fish and shellfish, beans, vegetables, fruits, miso, soy sauce, kelp, seaweed, and tea. Based on the results of sensitivity analysis in this study, the prevention strategy may add additional restricted use in cake and waffle, kelp, and snack. To reduce exposure, the Taiwan Department of Health and Welfare has twice announced (2014 and 2016) that only limited quantities of Al-containing food supplements may be used in specific seafood, pickled vegetables, fried foods, and pastries.

Any comparison study of Al in food will have some limitations. This is due to the variation in the sampling design, selected food, food processing (as different types of food additives containing aluminum used in food processing), and procedures for preparation, storage, and packaging of foodstuffs [45,46]. The EFSA states that cereals and cereal products, vegetables, beverages and certain infant formulae and others, appear to be the main contributors of Al intake [13].

In this study, we neither estimated the consumer only population for Al exposure nor included the exposure to Al from other sources, which could lead to an underestimation of total dietary exposure to Al. Moreover, in Zhou [26], we did not group the original 19 Al-rich food categories, into 12 categories. We did not adopt the other seven categories from Zhou [26], because these were adopted from other countries. Complementary to the studies assessing dietary Al exposure in Taiwan, three studies [17,21,25] gathered food representative of the diet and to matching with the major food groups consumed in the Taiwanese, such as rice, milk, eggs, vegetables, etc. We aimed to be as close as possible to consumer’s real dietary habits to accurately reflect dietary exposure to Al in Taiwanese.

On the other hand, the food sample data were based on a single 24 h recall and reported from years 2013, 2014, and 2016; however, the dietary consumption data were from 2017. The temporal disconnect between those data might be of concern and should be mentioned. Moreover, data on Al concentrations from two China studies [21,27] and food consumption rates used in this study did not achieve the most appropriate food matching. Different food characteristics such as processing and preparation of the food influence the food matching. For food matching issues, the United Nations Food and Agriculture Organization/International Network of Food Data Systems (FAO/INFOODS) has been working on harmonization, developing standards, guidelines, and tools. They recently developed guidelines, including on food identification, food components, recipes, and documentation for ensuring the quality of food matching studies [47].

Moreover, the analysis methods of Al concentration in food were different, as flame atomic absorption spectrometry method according to NIEA M103.00C and NIEA M111.00C [26], inductively coupled plasma mass spectrometry method [21,27], and inductively coupled plasma optical emission spectrometry method [25] were used.

## 5. Conclusions

This is the first study to present comprehensive and current data on estimations of dietary exposure to Al for the different age-sex groups among the general population in Taiwan. The EWIs declined with increasing age (7–12 > 13–15 > 16–18 > 19–64 > 65+). The EWIs of Al-rich food in cake + waffle, kelp, snacks, and bread contributed 20%, 17%, 17%, and 11%, respectively, to the total EWIs, which corresponds with the much higher consumption rates for these four foods. The 75th percentile of EWIs for the children aged 4–6 years had a %PTWI valued at over 100%, indicating a potential risk of Al intake to this age group via dietary exposure. Therefore, we suggest that should be a concern about the consumption of Al-rich foods by children in Taiwan.

## Figures and Tables

**Table 1:** Descriptive statistics for aluminum concentrations (mg/kg) of 12 aluminum-rich food items in Taiwan (adopted from Zhou [25] and Yeh et al. [26] and the aluminum concentrations (mg/kg) in nine additional food consumption items (adopted from Jiang et al. [21] and Yang et al. [27]. P_50_ and P_95_ indicate the 50th-percentile and 95th-percentile, respectively.

Food Items	Sample Size	Range	P50	P95	Mean	SD
1. Steamed pastry ^a^	17	ND–497.2	53.4	399.7	114	152.6
2. Steamed sponge cake ^a^	15	ND–628.1	230.3	456	217.3	169
3. Bread ^a^	13	ND–415.6	7.4	191.3	44.5	112.6
4. Cake ^a^*	12	ND–178.7	4.5	130.6	30.1	53.7
5. Waffle *	25	ND–535.2	27.3	485.7	132	177.4
6. Biscuits ^a^	14	ND–112.3	4.8	63.9	18.3	30
7. Fried bread stick ^a^	14	ND–608.1	462.1	607.3	440.9	150.1
8. Snacks ^a^	13	ND–414.6	31.4	232.2	67.5	110
9. Salted jellyfish body ^a^	10	ND–1389.6	785.8	1337.2	876.6	328.2
10. Kelp ^a^	10	ND–1239.5	12.4	1163.2	424.5	547.1
11. Sugar coated products ^b^	53	ND–94.7	39.5	88.7	45.7	37.7
12. Green bean noodle ^a^	5	ND–36.2	12	34.1	15.2	15.5
13. Rice ^c^	55	0.1–18.3	1.2	-	1.9	2.5
14. Milk ^c^	23	0.2–15.2	1.9	-	3.7	4.2
15. Eggs ^c^	23	0.4–4.2	1.8	-	2.1	1.1
16. Meat ^c^	23	0.3–26.8	4.0	-	6.1	7.2
17. Vegetables ^c^	284	0.0–61.3	2.6	-	4.8	6.4
18. Fish and seafood ^c^	74	0.0–122.7	1.2	-	4.1	15.2
19. Beans ^c^	23	3.2–48.5	15.4	-	18.7	14.7
20. Fruit ^c^	4	0.6–2.0	1.2	-	1.2	0.6
21. Drinking water ^d^	184	ND–0.118	0.052	-	-	-

ND: not detected. -: not estimated. ^a^ Food items and values adopted from Zhou [26]. ^b^ Food items and values adopted from Yeh et al. [25] (N = 51) and Zhou [26] (N=2). This study only cited the Al levels (N = 51) in chocolate (N = 13) and candies (N = 38) from the Yeh et al. [25] study. ^c^ Food items and values adopted from Jiang et al. [27]. ^d^ Food items and values adopted from Yang et al. [21]. * Food items 4 and 5 were merged. The geometric mean 16.32 and geometric standard deviation 2.51 were used as fitted parameters for log-normal distribution.

**Table 2:** Estimated weekly intake (EWI) (mg/kg-bw/wk) for all age-sex groups. Contribution percentages (%) for each food group are also presented. The definition of contribution percentages was calculated by “average EWI across age-sex subgroups” divided to “total EDI”.

Food Items	0–3		4–6		7–12		13–15		16–18		19–64		65+		Average across Age-Sex Subgroups	Percentage (%)	19–49
	F	M	F	M	F	M	F	M	F	M	F	M	F	M			F
1.Steamed pastry	0.028	0.003	0.064	0.034	0.027	0.031	0.018	0.036	0.011	0.012	0.033	0.037	0.051	0.148	0.038	3.56%	0.016
2.Steamed sponge cake	0.103	0.000	0.036	0.041	0.071	0.017	0.014	0.029	0.049	0.041	0.057	0.031	0.033	0.055	0.041	3.84%	0.064
3.Bread	0.097	0.123	0.259	0.160	0.166	0.172	0.144	0.122	0.132	0.101	0.057	0.057	0.029	0.027	0.118	10.99%	0.063
4.Cake(+5.Waffle)	0.236	0.590	0.333	0.517	0.225	0.207	0.183	0.112	0.147	0.119	0.124	0.077	0.057	0.067	0.214	19.97%	0.139
6.Biscuits	0.028	0.046	0.028	0.034	0.016	0.017	0.009	0.011	0.009	0.007	0.005	0.006	0.004	0.004	0.016	1.50%	0.005
7.Fried bread stick	0.024	0.000	0.000	0.001	0.044	0.038	0.111	0.057	0.125	0.117	0.032	0.008	0.077	0.000	0.045	4.23%	0.016
8.Snacks	0.477	0.400	0.451	0.360	0.203	0.144	0.084	0.081	0.113	0.073	0.040	0.033	0.016	0.017	0.178	16.62%	0.054
9.Salted jellyfish body	0.000	0.000	0.000	0.000	0.006	0.005	0.004	0.003	0.003	0.003	0.003	0.003	0.003	0.003	0.003	0.24%	0.001
10.Kelp	0.097	0.185	0.402	0.565	0.188	0.136	0.144	0.118	0.080	0.086	0.187	0.149	0.063	0.098	0.178	16.66%	0.193
11.Sugar coated products	0.059	0.044	0.040	0.031	0.020	0.019	0.022	0.017	0.014	0.010	0.010	0.009	0.002	0.002	0.021	1.99%	0.011
12.Green bean noodle	0.012	0.012	0.007	0.002	0.012	0.007	0.006	0.006	0.005	0.004	0.006	0.004	0.005	0.001	0.006	0.59%	0.007
13.Rice	0.094	0.118	0.094	0.119	0.062	0.085	0.049	0.068	0.035	0.060	0.037	0.059	0.055	0.068	0.072	6.69%	0.034
14.Milk	0.013	0.039	0.036	0.023	0.020	0.018	0.009	0.012	0.010	0.009	0.004	0.004	0.002	0.003	0.014	1.34%	0.005
15.Eggs	0.017	0.016	0.018	0.024	0.018	0.018	0.013	0.013	0.012	0.013	0.006	0.007	0.003	0.004	0.013	1.20%	0.006
16.Meat	0.039	0.038	0.074	0.075	0.058	0.081	0.049	0.060	0.043	0.055	0.031	0.041	0.015	0.021	0.049	4.55%	0.035
17.Vegetable	0.013	0.010	0.016	0.018	0.014	0.015	0.012	0.012	0.009	0.009	0.016	0.013	0.018	0.017	0.014	1.28%	0.015
18.Fish and seafood	0.017	0.019	0.024	0.027	0.016	0.014	0.011	0.012	0.007	0.008	0.013	0.014	0.009	0.014	0.015	1.37%	0.013
19.Beans	0.008	0.018	0.011	0.012	0.008	0.010	0.007	0.005	0.008	0.005	0.009	0.006	0.007	0.011	0.009	0.84%	0.008
20.Fruit	0.027	0.033	0.041	0.038	0.025	0.028	0.019	0.013	0.017	0.011	0.027	0.020	0.019	0.024	0.024	2.28%	0.025
21.Drinking water	0.013	0.013	0.002	0.002	0.001	0.001	0.000	0.001	0.000	0.001	0.001	0.001	0.002	0.002	0.003	0.26%	0.001
Total	1.404	1.708	1.937	2.083	1.198	1.060	0.909	0.788	0.830	0.742	0.697	0.578	0.471	0.585	1.071		0.713

## Supplementary Materials

The following are available online at: https://www.mdpi.com/1660-4601/18/3/1099/s1, Figure S1: Mean consumption rate (g/day) according to age-sex group for 12 aluminum-rich food items (adopted from Food Consumption Database in Taiwan, 2017) [28], Figure S2: Mean consumption rate (g/day) by age-sex groups for 9 additional food consumption items (adopted from Food Consumption Database in Taiwan, 2017) [28], Figure S3: Sensitivity analysis for estimated weekly intake (EWI) (mg/kg-bw/wk) for age-groups of 0–3, 4–6, 7–12 yrs, Figure S4: Sensitivity analysis for estimated weekly intake (EWI) (mg/kg-bw/wk) for age-groups of 13–15, 16–18, 19–64, and 65+ yrs, Table S1: Body weight (kg) for all age-sex groups., Table S2: Consumption rate for general population (g/day) of 12 aluminum-rich food items (adopted from Food Consumption Database in Taiwan, 2017) [28], Table S3: Consumption rate for general population (g/day) of 9 additional food consumption items (adopted from Food Consumption Database in Taiwan, 2017) [28], Table S4: Uncertainties of input parameter for the current exposure assessment.
