# Supplementary material for: Probabilistic Risk Analysis to Assess Dietary Exposure to Aluminum in the Taiwanese Population
Source: Int J Environ Res Public Health. 2021 Jan 26;18(3):1099. doi: 10.3390/ijerph18031099 (PMC7908212; doi:10.3390/ijerph18031099)
Supplement: Supplementary file 1 [file ijerph-18-01099-s001.pdf]

Table S1. Body weight (kg) for all age-sex groups.

Age groups	0–3		4–6		7–12		13–15		16–18		19–64		65+		19–49
Sex	F [a] (N=216)	M [a] (N=213)	F (N=231)	M (N=233)	F (N=411)	M (N=416)	F (N=688)	M (N=614)	F (N=641)	M (N=633)	F (N=1015)	M (N=1000)	F (N=447)	M (N=446)	F [b] (N=661)
Mean[b]	12.84	12.99	20.03	21.00	33.22	34.77	50.44	57.62	54.42	65.98	57.27	69.33	57.07	63.83	56.74
SD[b]	3.18	3.11	4.42	4.55	10.66	10.97	10.16	15.12	11.06	15.11	9.75	11.07	9.68	9.91	10.26

[a] M: male; F: female. [b] indicates childbearing age.
[b] The body weight for all age-sex groups was assumed a normal distribution with the mean and standard deviation.

Table S2. Consumption rate for general population (g/day) of 12 aluminum-rich food items (adopted from Food Consumption Database in Taiwan, 2017) [28].

Food items	Age groups	0–3		4–6		7–12		13–15		16–18		19–64		65+		19–49
	Sex	F [a] (N=216)	M [a] (N=213)	F (N=231)	M (N=233)	F (N=411)	M (N=416)	F (N=688)	M (N=614)	F (N=641)	M (N=633)	F (N=1015)	M (N=1000)	F (N=447)	M (N=446)	F [b] (N=661)
1.Steamed pastry	mean[b]	0.45	0.05	1.60	0.89	1.12	1.34	1.15	2.60	0.78	1.02	2.34	3.25	3.66	11.82	1.16
	SD[b]	4.97	0.61	14.72	6.97	11.82	9.47	15.71	19.50	10.76	10.70	12.79	13.29	23.30	54.35	8.47
2.Steamed sponge cake	mean[b]	0.87	0.00	0.48	0.57	1.54	0.38	0.46	1.10	1.74	1.78	2.13	1.40	1.24	2.32	2.38
	SD[b]	9.76	0.00	5.02	5.33	14.51	5.46	5.10	13.82	13.99	11.85	17.28	13.90	9.87	20.17	16.50
3.Bread	mean[b]	4.02	5.13	16.69	10.80	17.73	19.24	23.26	22.63	23.15	21.34	10.50	12.67	5.38	5.57	11.43
	SD[b]	18.11	23.72	42.74	32.09	65.72	57.55	63.62	67.17	67.98	63.33	38.38	46.02	24.78	24.81	37.07
4.Cake(+5.Waffle)	mean[b]	5.35	13.51	11.77	19.12	13.17	12.67	16.30	11.33	14.09	13.81	12.53	9.40	5.74	7.55	13.93
	SD[b]	29.48	67.07	30.01	176.64	57.16	50.39	42.74	63.76	64.84	130.74	43.02	55.88	25.20	32.61	42.65
6.Biscuits	mean[b]	2.84	4.67	4.32	5.56	4.11	4.61	3.68	5.03	3.64	3.52	2.24	3.30	1.90	1.87	2.42
	SD[b]	11.95	23.78	26.34	21.41	21.39	28.91	24.14	34.27	17.96	20.55	15.02	20.72	11.43	15.74	14.39
7.Fried bread stick	mean[b]	0.10	0.00	0.00	0.01	0.47	0.43	1.82	1.06	2.20	2.50	0.60	0.18	1.42	0.00	0.30
	SD[b]	1.36	0.00	0.06	0.15	4.48	3.62	29.23	12.01	16.66	24.15	10.09	2.43	24.01	0.07	4.30
8.Snacks	mean[b]	12.97	11.00	19.09	15.98	14.29	10.56	8.99	9.90	13.00	10.14	4.80	4.80	1.97	2.25	6.45
	SD[b]	45.62	79.00	63.97	84.53	96.94	49.49	47.68	71.85	69.39	64.69	49.80	49.80	18.89	30.30	48.98
9.Salted jellyfish body [c]	mean	0.00	0.00	0.00	0.00	0.03	0.03	0.03	0.03	0.03	0.03	0.03	0.03	0.03	0.03	0.01
10.Kelp	mean[b]	0.42	0.81	2.71	3.99	2.10	1.59	2.44	2.29	1.46	1.90	3.61	3.47	1.21	2.10	3.69
	SD[b]	3.92	10.05	17.42	22.19	8.75	5.38	11.53	9.14	7.42	10.72	21.96	20.79	10.46	15.30	22.22
11.Sugar coated products	mean[b]	2.36	1.79	2.53	2.03	2.12	2.06	3.50	3.13	2.43	2.03	1.77	1.90	0.31	0.37	2.04
	SD[b]	12.40	27.74	28.00	21.41	25.33	33.29	27.53	40.30	18.18	19.63	17.30	20.62	13.01	11.21	16.32
12.Green bean noodle	mean[b]	1.45	1.46	1.28	0.40	3.59	2.36	3.02	3.42	2.48	2.36	3.33	2.42	2.45	0.47	3.70
	SD[b]	10.32	9.25	7.95	3.77	31.20	24.29	17.80	28.14	18.22	15.98	19.49	25.55	25.18	4.74	19.72
**SUM (1-12)**	**mean**	**30.83**	**38.42**	**60.47**	**59.34**	**60.27**	**55.27**	**64.65**	**62.52**	**64.99**	**60.42**	**43.88**	**42.82**	**25.31**	**34.35**	**47.51**

[a] M: male; F: female. [b] indicates childbearing age. [c] Consumption rates of salted jellyfish body were adopted from Zhou (2014) [26].
[b] The consumption rate of 12 aluminum-rich food items was assumed a log-normal distribution with the geometric mean and geometric standard deviation.

Table S3. Consumption rate for general population (g/day) of 9 additional food consumption items (adopted from Food Consumption Database in Taiwan, 2017) [28]

**Food items**	**Age groups**	**0–3**		**4–6**		**7–12**		**13–15**		**16–18**		**19–64**		**65+**		**19–49**
	**Sex**	**F [a] (N=216)**	**M [a] (N=213)**	**F (N=231)**	**M (N=233)**	**F (N=411)**	**M (N=416)**	**F (N=688)**	**M (N=614)**	**F (N=641)**	**M (N=633)**	**F (N=1015)**	**M (N=1000)**	**F (N=447)**	**M (N=446)**	**F [b] (N=661)**
13.Rice	mean[b]	90.91	115.41	141.79	187.42	155.21	221.41	186.92	296.51	143.56	298.37	157.45	305.49	236.70	325.83	143.51
	SD[b]	123.54	182.98	122.74	156.29	213.12	214.16	230.64	252.83	179.60	241.10	213.74	467.22	296.57	342.92	180.21
14.Milk	mean[b]	6.43	19.58	27.47	18.57	25.84	23.56	17.48	25.91	21.55	23.77	9.58	10.42	3.87	7.38	11.57
	SD[b]	72.49	223.70	98.38	90.30	144.33	92.45	73.94	120.88	123.47	124.71	75.09	80.45	40.44	62.48	79.29
15.Eggs	mean[b]	14.74	14.40	25.09	34.06	39.66	41.55	44.33	50.80	43.13	57.34	22.71	33.79	9.72	16.21	24.12
	SD[b]	46.32	29.10	36.39	57.64	54.67	74.52	75.49	84.25	66.06	71.97	40.68	37.01	27.42	32.75	40.49
16.Meat	mean[b]	11.80	11.60	34.65	36.71	44.94	66.17	57.78	81.19	55.20	85.50	41.51	67.29	20.55	32.05	46.61
	SD[b]	13.76	13.78	37.42	41.38	48.15	69.18	60.57	84.76	57.06	88.58	45.45	73.56	24.59	38.43	50.20
17.Vegetable	mean[b]	4.80	4.00	9.55	11.49	13.77	15.20	17.33	20.19	14.89	17.59	26.76	25.85	30.90	32.93	24.69
	SD[b]	15.85	16.90	34.16	51.76	58.20	76.25	62.68	54.73	57.49	59.10	102.02	104.46	122.28	123.09	87.08
18.Fish and seafood	mean[b]	7.71	8.82	16.58	20.12	18.08	16.40	20.09	24.25	13.58	18.26	25.77	33.47	18.12	32.05	25.99
	SD[b]	29.60	23.13	52.20	46.17	41.06	36.22	74.07	74.57	29.74	42.91	80.82	110.80	58.11	45.22	68.42
19.Beans	mean[b]	0.76	1.82	1.67	1.98	2.14	2.66	2.58	2.28	3.40	2.57	3.81	3.43	2.87	5.26	3.61
	SD[b]	4.32	11.54	6.84	7.41	8.81	12.65	17.21	21.25	13.58	11.29	16.76	13.96	14.18	31.70	14.80
20.Fruit	mean[b]	41.92	50.32	98.66	95.98	97.74	115.15	112.75	85.94	107.26	86.01	182.85	168.19	132.35	181.17	169.12
	SD[b]	82.75	101.74	157.84	164.91	173.49	252.40	220.11	196.93	239.85	222.60	232.14	282.11	197.44	308.31	203.28
21.Drinking water	mean[b]	456.7	457.77	104.94	136.26	67.84	73.25	55.3	81.91	73.93	104.28	171.12	168.85	280.96	263.19	157.86
	SD[b]	379.79	352.02	250.68	267.90	307.09	389.40	150.14	359.19	362.17	381.77	338.46	559.38	619.06	400.08	307.63
**SUM (13-21)**	**mean**	**666.6**	**722.14**	**520.87**	**601.93**	**525.49**	**630.62**	**579.21**	**731.5**	**541.49**	**754.11**	**685.44**	**859.6**	**761.35**	**930.42**	**654.59**

[a] M: male; F: female. [b] indicates childbearing age.

[b] The consumption rate of 9 additional food consumption items was assumed a log-normal distribution with the geometric mean and geometric standard deviation.

Table S4. Uncertainties of input parameter for the current exposure assessment.

Sources of uncertainty	Direction[a]
Food consumption database: 24-hour recall data, food items mismatching, and survey years	↓↑
Concentration data: 12 Al-rich food items, high consumption food items	↓
Limited sample size of concentration of some food items	↓
Assumed distribution models of consumption rate and concentration of food items	↑↓

[a] ↑Potential over-estimation of risk or exposure; ↓ Potential under-estimation of risk or exposure.

Appendix Figure captions

Fig. **S1.** Mean consumption rate (g/day) according to age-sex group for 12 aluminum-rich food items (adopted from Food Consumption Database in Taiwan, 2017) [28].

Fig. **S2.** Mean consumption rate (g/day) by age-sex groups for 9 additional food consumption items (adopted from Food Consumption Database in Taiwan, 2017) [28].

Fig. **S3.** Sensitivity analysis for estimated weekly intake (EWI) (mg/kg-bw/wk) for age-groups of 0–3, 4–6, 7–12 yrs.

Fig. **S4.** Sensitivity analysis for estimated weekly intake (EWI) (mg/kg-bw/wk) for age-groups of 13–15, 16–18, 19–64, and 65+ yrs.

Fig. S1.

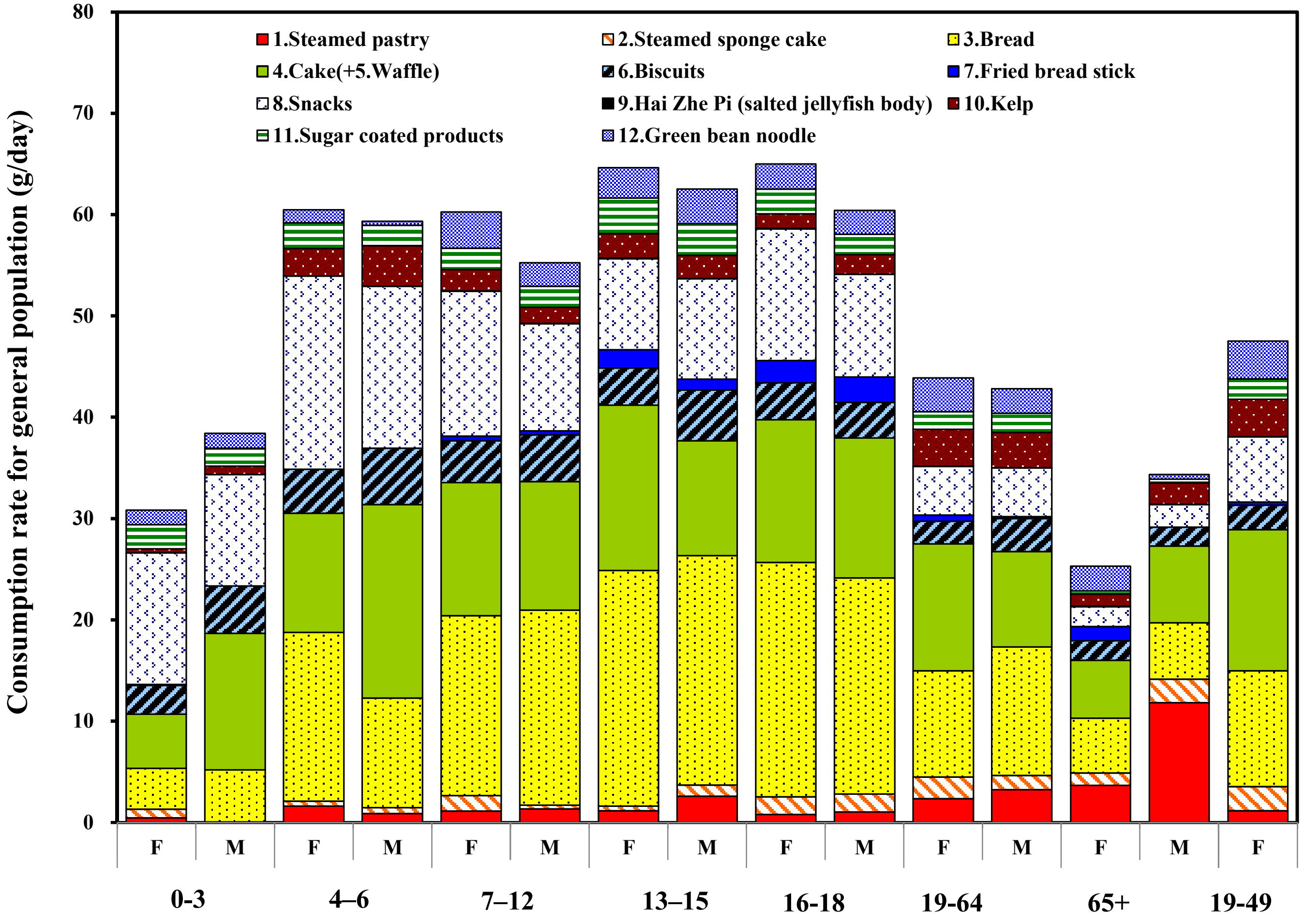
Consumption rate for general population (g/day)
1.Steamed pastry
2.Steamed sponge cake
3.Bread
4.Cake(+5.Waffle)
6.Biscuits
7.Fried bread stick
8.Snacks
9.Hai Zhe Pi (salted jellyfish body)
10.Kelp
11.Sugar coated products
12.Green bean noodle
0
10
20
30
40
50
60
70
80
F
M
F
M
F
M
F
M
F
M
F
M
F
M
F
0-3
4–6
7–12
13–15
16-18
19-64
65+
19-49

Fig. S2.

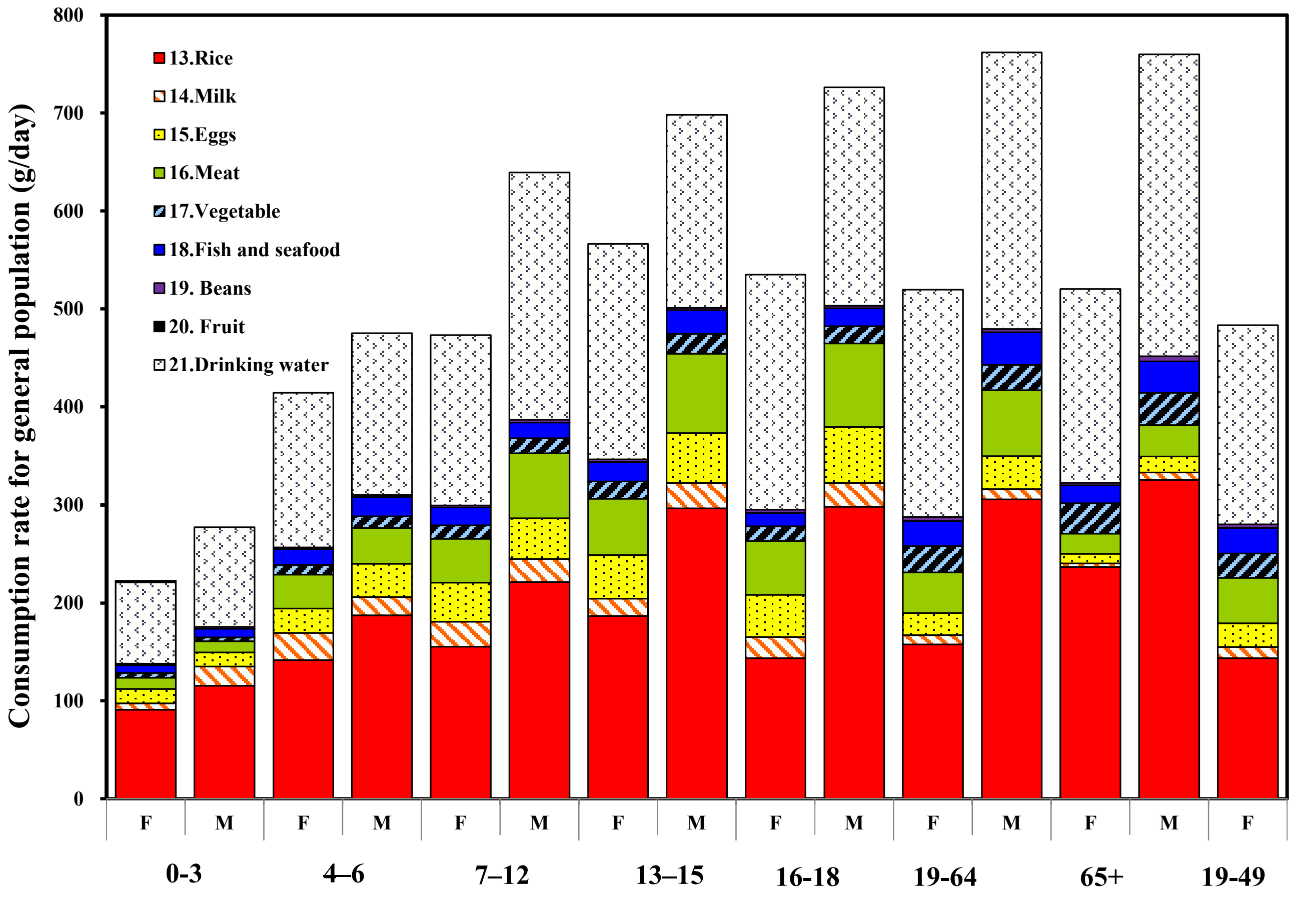
800
700
600
500
400
300
200
100
0
Consumption rate for general population (g/day)
13.Rice
14.Milk
15.Eggs
16.Meat
17.Vegetable
18.Fish and seafood
19. Beans
20. Fruit
21.Drinking water
F
M
F
M
F
M
F
M
F
M
F
M
F
M
F
0-3
4–6
7–12
13–15
16-18
19-64
65+
19-49

Fig. S3.

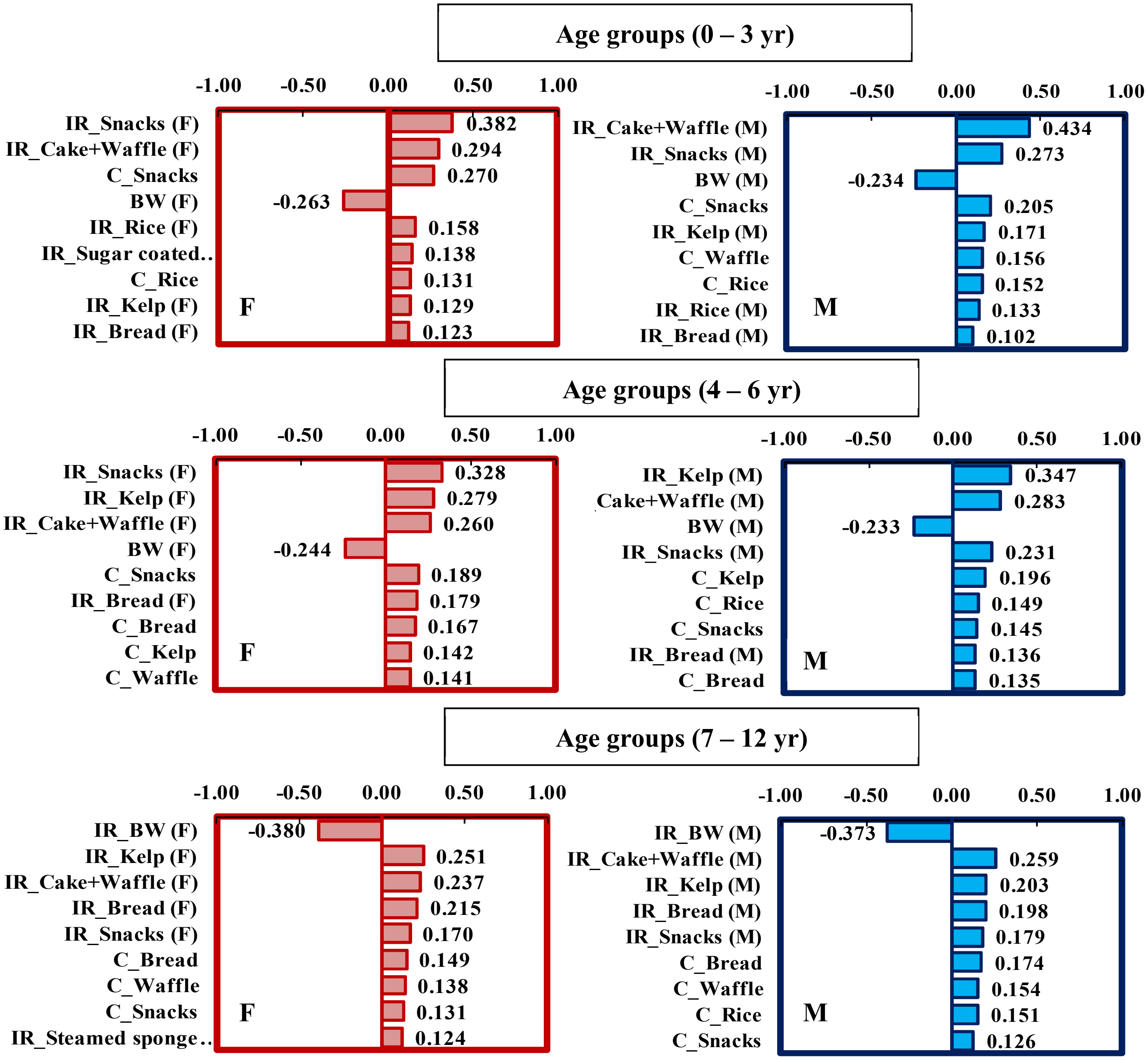
Age groups (0 – 3 yr)
-1.00 -0.50 0.00 0.50 1.00
IR_Snacks (F) 0.382
IR_Cake+Waffle (F) 0.294
C_Snacks 0.270
BW (F) -0.263
IR_Rice (F) 0.158
IR_Sugar coated.. 0.138
C_Rice 0.131
IR_Kelp (F) 0.129
IR_Bread (F) 0.123
F
IR_Cake+Waffle (M) 0.434
IR_Snacks (M) 0.273
BW (M) -0.234
C_Snacks 0.205
IR_Kelp (M) 0.171
C_Waffle 0.156
C_Rice 0.152
IR_Rice (M) 0.133
IR_Bread (M) 0.102
M
Age groups (4 – 6 yr)
IR_Snacks (F) 0.328
IR_Kelp (F) 0.279
IR_Cake+Waffle (F) 0.260
BW (F) -0.244
C_Snacks 0.189
IR_Bread (F) 0.179
C_Bread 0.167
C_Kelp 0.142
C_Waffle 0.141
F
IR_Kelp (M) 0.347
Cake+Waffle (M) 0.283
BW (M) -0.233
IR_Snacks (M) 0.231
C_Kelp 0.196
C_Rice 0.149
C_Snacks 0.145
IR_Bread (M) 0.136
C_Bread 0.135
M
Age groups (7 – 12 yr)
IR_BW (F) -0.380
IR_Kelp (F) 0.251
IR_Cake+Waffle (F) 0.237
IR_Bread (F) 0.215
IR_Snacks (F) 0.170
C_Bread 0.149
C_Waffle 0.138
C_Snacks 0.131
IR_Steamed sponge.. 0.124
F
IR_BW (M) -0.373
IR_Cake+Waffle (M) 0.259
IR_Kelp (M) 0.203
IR_Bread (M) 0.198
IR_Snacks (M) 0.179
C_Bread 0.174
C_Waffle 0.154
C_Rice 0.151
C_Snacks 0.126
M

Fig. S4.

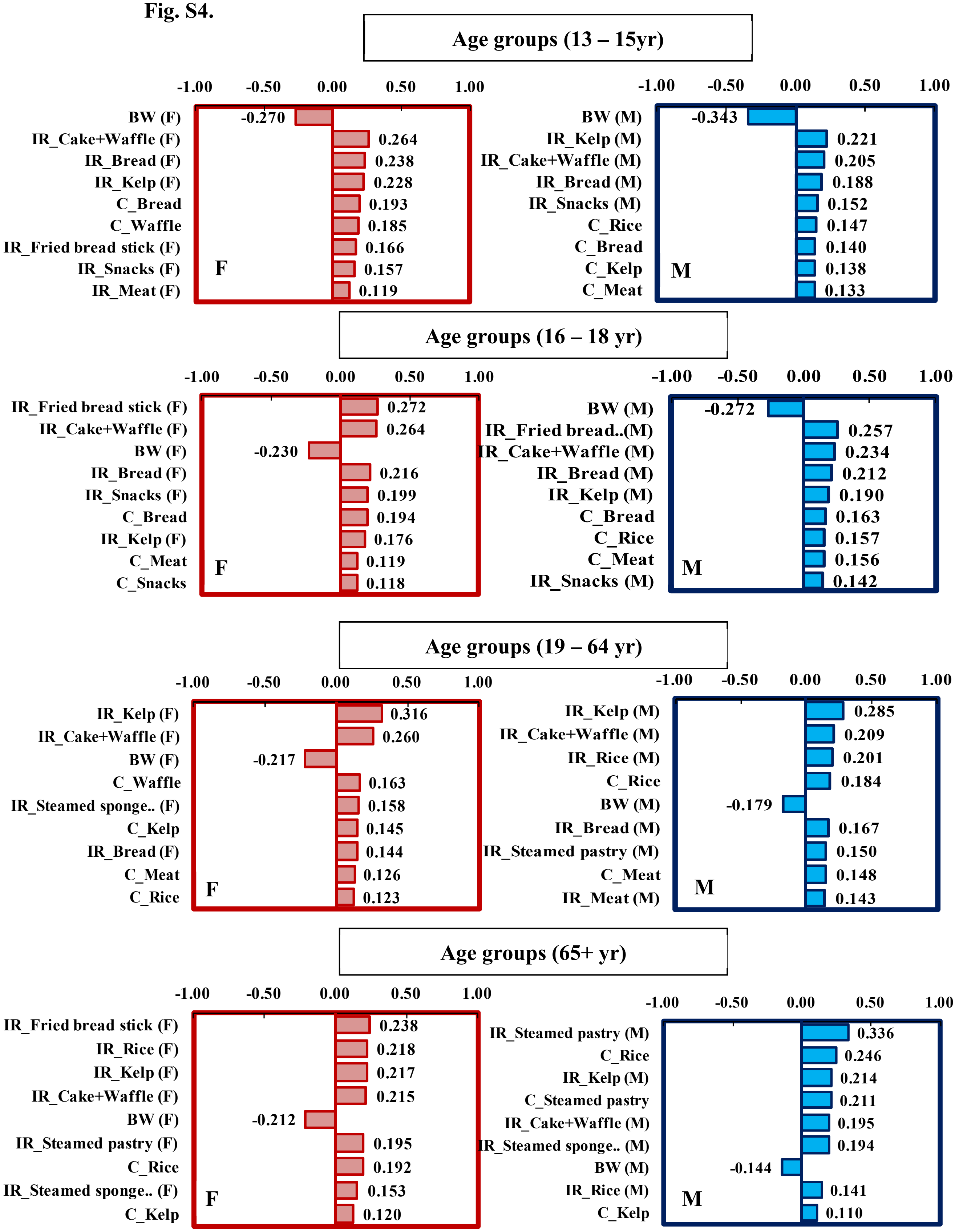
Age groups (13 – 15yr)
-1.00 -0.50 0.00 0.50 1.00
F
BW (F) -0.270
IR_Cake+Waffle (F) 0.264
IR_Bread (F) 0.238
IR_Kelp (F) 0.228
C_Bread 0.193
C_Waffle 0.185
IR_Fried bread stick (F) 0.166
IR_Snacks (F) 0.157
IR_Meat (F) 0.119
M
BW (M) -0.343
IR_Kelp (M) 0.221
IR_Cake+Waffle (M) 0.205
IR_Bread (M) 0.188
IR_Snacks (M) 0.152
C_Rice 0.147
C_Bread 0.140
C_Kelp 0.138
C_Meat 0.133
Age groups (16 – 18 yr)
F
IR_Fried bread stick (F) 0.272
IR_Cake+Waffle (F) 0.264
BW (F) -0.230
IR_Bread (F) 0.216
IR_Snacks (F) 0.199
C_Bread 0.194
IR_Kelp (F) 0.176
C_Meat 0.119
C_Snacks 0.118
M
BW (M) -0.272
IR_Fried bread..(M) 0.257
IR_Cake+Waffle (M) 0.234
IR_Bread (M) 0.212
IR_Kelp (M) 0.190
C_Bread 0.163
C_Rice 0.157
C_Meat 0.156
IR_Snacks (M) 0.142
Age groups (19 – 64 yr)
F
IR_Kelp (F) 0.316
IR_Cake+Waffle (F) 0.260
BW (F) -0.217
C_Waffle 0.163
IR_Steamed sponge.. (F) 0.158
C_Kelp 0.145
IR_Bread (F) 0.144
C_Meat 0.126
C_Rice 0.123
M
IR_Kelp (M) 0.285
IR_Cake+Waffle (M) 0.209
IR_Rice (M) 0.201
C_Rice 0.184
BW (M) -0.179
IR_Bread (M) 0.167
IR_Steamed pastry (M) 0.150
C_Meat 0.148
IR_Meat (M) 0.143
Age groups (65+ yr)
F
IR_Fried bread stick (F) 0.238
IR_Rice (F) 0.218
IR_Kelp (F) 0.217
IR_Cake+Waffle (F) 0.215
BW (F) -0.212
IR_Steamed pastry (F) 0.195
C_Rice 0.192
IR_Steamed sponge.. (F) 0.153
C_Kelp 0.120
M
IR_Steamed pastry (M) 0.336
C_Rice 0.246
IR_Kelp (M) 0.214
C_Steamed pastry 0.211
IR_Cake+Waffle (M) 0.195
IR_Steamed sponge.. (M) 0.194
BW (M) -0.144
IR_Rice (M) 0.141
C_Kelp 0.110
